# Ferroptosis and bone metabolic diseases: the dual regulatory role of the Nrf2/HO-1 signaling axis

**DOI:** 10.3389/fcell.2025.1615197

**Published:** 2025-09-05

**Authors:** Wei Nan, Wen-Ming Zhou, Jian-Lan Zi, Yong-Qiang Shi, Yan-Bo Dong, Wei Song, Yan-Chao Ma, Hai-Hong Zhang

**Affiliations:** ^1^ The Second Hospital & Clinical Medical School, Lanzhou University, Lanzhou, China; ^2^ Orthopaedics Key Laboratory of Gansu Province, Lanzhou, China; ^3^ The People’s Hospital Of Chuxiong Yi Autonomous Prefecture, Chuxiong, China

**Keywords:** Nrf2, HO-1, ferroptosis, bone homeostasis, oxidative stress

## Abstract

Ferroptosis, an iron-dependent form of regulated cell death characterized by lipid peroxidation, has emerged as a pivotal mechanism in bone disorders including osteoporosis and osteonecrosis. The nuclear factor erythroid 2–related factor 2 (Nrf2)/heme oxygenase-1 (HO-1) signaling axis plays a paradoxical role—contributing to cytoprotection under oxidative stress, yet potentially promoting ferroptosis through excessive iron accumulation. This review summarizes how the Nrf2/HO-1 pathway modulates ferroptosis across osteoblasts, osteoclasts, and osteocytes, and its impact on bone homeostasis. We explore the pathway’s involvement in the shift from physiological bone remodeling to pathological bone loss. Given its dual role, the Nrf2/HO-1 axis represents both a challenge and an opportunity for therapeutic intervention. Understanding its context-specific functions is essential for developing precise, ferroptosis-targeted strategies in bone disease treatment.

## 1 Introduction

Bone homeostasis is a fundamental physiological process that preserves the structural integrity and mechanical function of bone tissue. This process is primarily governed by the dynamic balance between osteoblasts and osteoclasts (Zhu et al., 2024; Zhang et al., 2021). Osteoblasts are responsible for the synthesis and mineralization of new bone, while osteoclasts facilitate bone remodeling by resorbing aged or damaged bone tissue (Wu et al., 2024). Under normal physiological conditions, bone formation and resorption are tightly coupled to maintain bone mass and microarchitectural stability (Ze et al., 2025). However, this equilibrium is highly sensitive to the bone microenvironment and is regulated by various factors, including growth factors, cytokines, mechanical loading, oxidative stress, and metabolic byproducts (Gheorghe et al., 2024). Once the regulatory network is disordered, it can easily lead to abnormal bone metabolism, manifested as bone loss, bone microstructure destruction and decreased biomechanical properties.

In clinical settings, bone homeostasis imbalance is closely associated with several metabolic bone disorders, most notably osteoporosis. The core pathological mechanism of osteoporosis involves an increased rate of bone resorption relative to bone formation, leading to trabecular microfracture, decreased bone mineral density (BMD), and a heightened risk of fracture (Sun et al., 2023; Tao et al., 2020). In recent years, osteonecrosis, particularly in the context of vertebral compression fractures, has drawn increasing attention. Studies suggest that impaired local blood supply, elevated oxidative stress, and osteocyte dysfunction post-fracture may contribute to osteocyte death and subsequent bone tissue necrosis, thereby impeding bone regeneration and repair (Cabrera et al., 2022; Li et al., 2023a). Furthermore, conditions such as stress-related bone injury, hormone-induced osteonecrosis, and chemotherapy-associated osteotoxicity are frequently accompanied by varying degrees of bone homeostasis disruption. However, the underlying mechanisms remain poorly understood and warrant systematic investigation at the cellular and molecular levels.

Cell death plays a pivotal role in both maintaining bone homeostasis and contributing to bone pathology. While earlier research has focused on classical cell death pathways such as apoptosis (Yao et al., 2023) and autophagy (Montaseri et al., 2020; Laha et al., 2022), recent attention has turned toward ferroptosis (Jiang et al., 2024)—a distinct, non-apoptotic form of programmed cell death characterized by iron-dependent lipid peroxidation. Ferroptosis is primarily driven by the inactivation of glutathione peroxidase 4 (GPX4), dysregulation of intracellular iron homeostasis, and excessive accumulation of reactive oxygen species (ROS), ultimately resulting in lipid membrane rupture and loss of cellular function (Wang et al., 2021). Although ferroptosis has been implicated in tumorigenesis, neurodegeneration, and cardiovascular diseases, its role in bone homeostasis, particularly in regulating osteoblast survival and function, remains inadequately understood.

Among the various signaling pathways that regulate ferroptosis, the Nrf2 and its downstream effector HO-1 constitute a key axis for oxidative stress defense, iron metabolism, and ROS detoxification (Wang et al., 2022). Under oxidative stress, Nrf2 dissociates from Keap1 repression, translocates to the nucleus, and induces the expression of a suite of antioxidant and iron-handling genes, including HO-1 (Guo et al., 2021). HO-1 catabolizes heme into ferrous iron (Fe^2+^), carbon monoxide (CO), and biliverdin (BV), thereby exerting cytoprotective effects under certain physiological conditions (Laporte et al., 2019). However, the Fe^2+^ released in this process may also exacerbate lipid peroxidation and trigger ferroptosis under pathological conditions, suggesting that the Nrf2/HO-1 pathway may play a dual regulatory role in determining osteocyte fate. On one hand, this pathway can suppress ferroptosis and protect osteoblast function by mitigating lipid ROS accumulation and preserving iron homeostasis (Iseda et al., 2022). On the other hand, persistent activation of this axis under certain stimuli may promote ferroptosis due to iron overload, impair bone formation, and ultimately disrupt bone homeostasis (Liu G. Z. et al., 2021).

Therefore, elucidating the precise regulatory mechanisms of the Nrf2/HO-1 pathway in osteocyte ferroptosis has significant implications for understanding the pathogenesis of bone metabolic diseases and identifying novel therapeutic targets. This review aims to comprehensively examine the role of ferroptosis in bone homeostasis, with a particular focus on the bidirectional effects of the Nrf2/HO-1 signaling pathway in osteocyte ferroptosis. Furthermore, it explores the current research progress and therapeutic prospects of targeting this pathway in conditions such as osteoporosis and vertebral osteonecrosis, thereby providing a theoretical foundation for future studies and precision medicine approaches in bone metabolic disorders.

## 2 The Nrf2/HO-1 pathway: structure and function

### 2.1 Structure and function of Nrf2

Nuclear factor erythroid 2–related factor 2 (Nrf2) is a pivotal member of the Cap ‘n’ Collar (CNC) family of transcription factors. As a key regulator of the cellular antioxidant defense system, Nrf2 exerts its biological functions through the coordinated action of multiple functional domains (Karunatilleke et al., 2021). Among these, the Neh2 domain, located at the N-terminus, is critically involved in the interaction with Kelch-like ECH-associated protein 1 (Keap1), which facilitates the ubiquitination and proteasomal degradation of Nrf2 under basal conditions (Wang M. et al., 2019). In contrast, the C-terminal basic leucine zipper (bZIP) domain is essential for DNA binding and transcriptional activation (Zhang S. et al., 2022).

Under physiological (non-stressed) conditions, Keap1—primarily localized in the cytoplasm—acts as a major negative regulator of Nrf2. It forms a part of an E3 ubiquitin ligase complex, with Cullin3 (Cul3) serving as the scaffold protein, promoting continuous ubiquitin-dependent degradation of Nrf2 and thereby maintaining its low basal expression (Alonso-Piñeiro et al., 2021). However, upon exposure to oxidative stress, electrophilic agents, or metabolic perturbations, key cysteine residues in Keap1 (notably Cys151, Cys273, and Cys288) undergo oxidation or covalent modification. These modifications induce conformational changes in Keap1, impairing its ability to bind and target Nrf2 for degradation (Pribil Pardun et al., 2024).

As a result, stabilized Nrf2 accumulates in the cytoplasm and translocates into the nucleus, where it forms heterodimers with small Maf proteins. This complex specifically binds to the antioxidant response element (ARE) within the promoter regions of target genes, thereby initiating the transcriptional activation of a wide array of cytoprotective genes, including heme oxygenase-1 (HO-1), NAD(P)H quinone dehydrogenase 1 (NQO1), glutamate–cysteine ligase modifier subunit (GCLM), and ferritin heavy chain 1 (FTH1), among others (Zhai et al., 2022). Taken together, Nrf2 is widely regarded as the master regulator of intracellular redox homeostasis. Its activation plays a central role in orchestrating antioxidant responses, regulating metal ion metabolism, and suppressing various forms of regulated cell death—including ferroptosis—thereby maintaining cellular integrity under stress conditions.

### 2.2 HO-1: Nrf2 downstream target genes and their metabolites function

Heme oxygenase-1 (HO-1) is one of the most prominent target genes in the Nrf2 transcriptional regulatory network (Song and Long, 2020). It encodes a rate-limiting enzyme responsible for the degradation of heme into three key metabolites: carbon monoxide (CO), ferrous iron (Fe^2+^), and biliverdin (BV). Biliverdin can subsequently be converted into bilirubin (BR) by the enzyme biliverdin reductase (Li et al., 2023b).

These metabolites—CO, Fe^2+^, and BV—exert significant pleiotropic effects in maintaining cellular homeostasis and modulating stress responses. As an intracellular signaling molecule, CO has anti-apoptotic, anti-inflammatory, and vasoregulatory properties (Wang et al., 2020). Meanwhile, BV and its derivative BR are fat-soluble antioxidants that can efficiently scavenge peroxides within the cell membrane’s phospholipid bilayer, thus mitigating oxidative damage (Xie F. et al., 2023). In terms of iron metabolism, the proteins ferritin, ferroportin (FPN), and transferrin receptor 1 (TfR1) constitute the iron homeostasis system, ensuring the safe transport, storage, and regulation of intracellular iron levels (Bogdan et al., 2016).

However, under conditions of sustained high expression of HO-1 or when iron regulation is impaired, excessive accumulation of Fe^2+^ can exacerbate oxidative stress. This is due to the Fenton reaction, where Fe^2+^ catalyzes the generation of highly reactive hydroxyl radicals (•OH), promoting lipid peroxidation and acting as a key driver of ferroptosis (Chen B. et al., 2024).

Thus, HO-1 exhibits a dose-dependent or context-dependent dual regulatory role in cellular stress defense and ferroptosis induction. Moderate activation of HO-1 confers protective effects against cellular damage, while excessive activation or dysregulation of downstream pathways can transform HO-1 into a pathogenic factor, contributing to cellular dysfunction and ferroptotic cell death.

### 2.3 Multiple biological functions of Nrf2/HO-1 pathway

**FIGURE 1:**
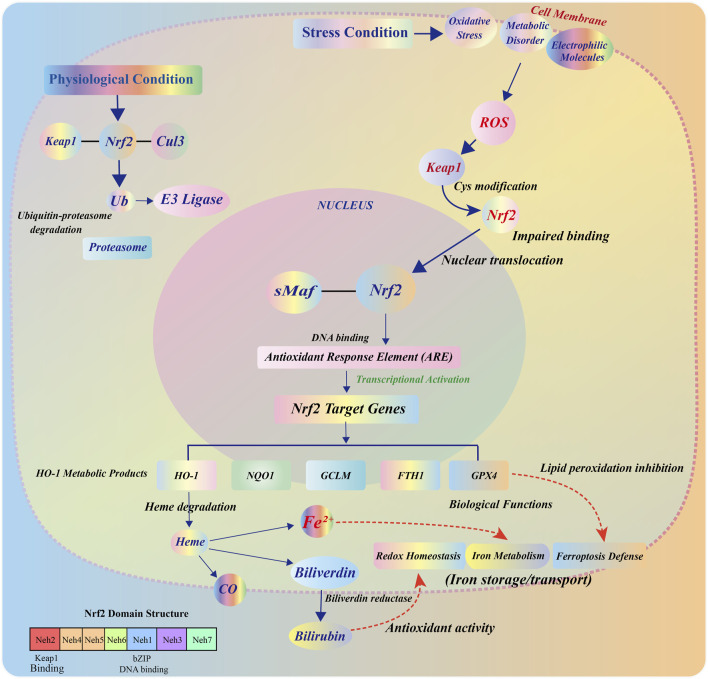
Structural composition, regulatory mechanism, and functional effects of the Nrf2/HO-1 pathway. Under steady-state conditions, Nrf2 is localized in the cytoplasm and forms a complex with Keap1, which binds to the Cul3-E3 ubiquitin ligase complex through the BTB, IVR, and DC domains. This complex mediates the ubiquitination and degradation of Nrf2 in the proteasome, maintaining low levels of its expression. Upon oxidative stress, cysteine residues in Keap1 undergo oxidative modification, allowing Nrf2 to escape degradation and accumulate in the cytoplasm. Nrf2 then translocates to the nucleus, forms a heterodimer with small Maf proteins, binds to the ARE, and induces the transcription of downstream target genes. As an important target gene of Nrf2, HO-1 encodes a heme-degrading enzyme that catalyzes the cleavage of heme into CO, Fe^2+^, and BV, which is converted to BR by biliverdin reductase. The released Fe^2+^ participates in the regulation of cellular iron homeostasis through FTH/FTL, FPN, and TfR1, and collectively contributes to antioxidant defense and ferroptosis inhibition.

The Nrf2/HO-1 signaling pathway is not only a central axis of the cellular antioxidant response but also plays a pivotal role in the regulation of several fundamental physiological processes. It orchestrates a tightly coordinated cytoprotective network that includes the following core functions: (1) Maintenance of Redox Homeostasis: Nrf2 mitigates the accumulation of ROS by upregulating a range of antioxidant enzymes, including HO-1, glutathione synthetase, superoxide dismutase (SOD), and catalase (CAT). Through this regulation, Nrf2 prevents mitochondrial dysfunction, DNA fragmentation, and protein oxidation, serving as the first line of defense against oxidative stress-induced cellular injury (Bu et al., 2023). (2) Iron Metabolism Remodeling and Homeostatic Regulation: Nrf2 modulates the expression of multiple genes involved in iron metabolism, such as ferritin, ferroportin (FPN), transferrin receptor 1 (TfR1), and hepcidin. These genes work in concert with Fe^2+^ released via HO-1-mediated heme degradation to maintain iron homeostasis (Kajarabille and Latunde-Dada, 2019). This regulatory mechanism is essential in protecting cells from iron overload-induced cytotoxicity, particularly under conditions of high metabolic activity or pathological iron accumulation. (3) Lipid Peroxidation Defense and Ferroptosis Suppression: In addition to its role in redox and iron balance, Nrf2 also regulates the expression of lipid peroxidation defense genes, such as glutathione peroxidase 4 (GPX4), ferritin heavy chain 1 (FTH1), and SLC7A11. These factors are essential for suppressing lipid ROS accumulation, preserving membrane integrity, and thereby inhibiting ferroptosis (Zhang et al., 2025). Notably, Nrf2 deficiency is often associated with GPX4 downregulation and increased phospholipid-derived ROS, which are critical initiating events in the ferroptotic cascade (Liao et al., 2024) (Figure 1).

Therefore, the Nrf2/HO-1 pathway serves as an integrated protective mechanism in response to various cellular stressors, regulating redox homeostasis, iron balance, and lipid peroxidation networks in a synergistic manner. However, its biological function exhibits significant tissue-specific and pathology-dependent variations. Some studies indicate that, under certain stimuli, this pathway may shift from a protective to a disease-promoting role, highlighting the complexity of its regulatory mechanisms.

## 3 Ferroptosis and its emerging role in bone homeostasis

This section comprehensively examines ferroptosis from two perspectives: first, we elucidate the molecular mechanisms underlying this unique form of regulated cell death; second, we explore how ferroptosis differentially affects various bone cell types and its implications for bone homeostasis.

### 3.1 Molecular mechanisms of ferroptosis

#### 3.1.1 The concept and molecular characteristics of ferroptosis

Ferroptosis is a distinct form of programmed cell death characterized by the iron-dependent accumulation of lipid peroxides, and it exhibits molecular and morphological features that are fundamentally different from those of apoptosis, autophagy, and necrosis (Ji et al., 2022; Sun et al., 2024). The hallmark of ferroptosis is the oxidation of polyunsaturated fatty acid (PUFA) residues in the phospholipid bilayer of the cell membrane. Morphologically, it is typically accompanied by reduced mitochondrial volume, loss of cristae, and diminished membrane potential, while the overall integrity of the plasma membrane remains largely unaffected (Xie Y. et al., 2023; Kim et al., 2023). Unlike apoptosis, ferroptosis does not involve the activation of caspase family proteins, but is instead driven by lipid ROS accumulation and GPX4 dysfunction (Zhong et al., 2022).

#### 3.1.2 The main molecular mechanisms of ferroptosis

**FIGURE 2:**
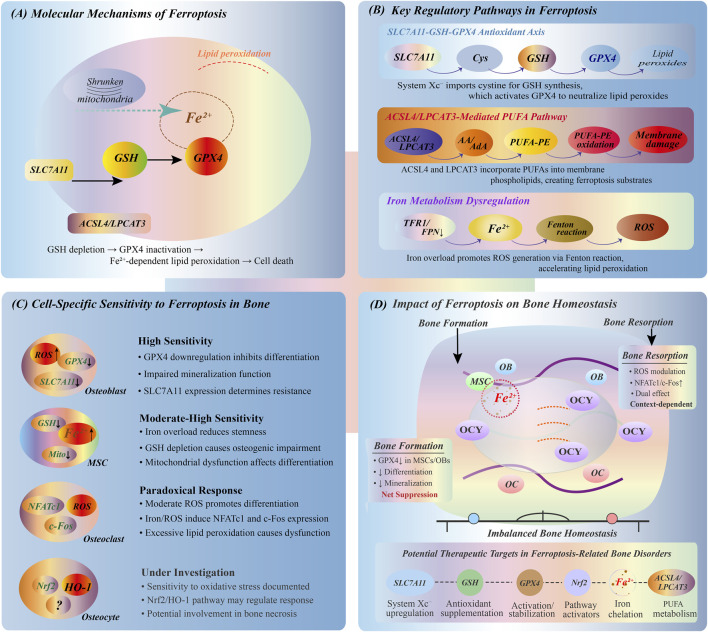
Ferroptosis mechanisms and bone cell responses. **(A)** Core ferroptosis mechanism showing Fe^2+^-dependent lipid peroxidation, mitochondrial dysfunction, and GPX4 inactivation leading to cell death. **(B)** Three key regulatory pathways: SLC7A11-GSH-GPX4 antioxidant axis for lipid peroxide removal; ACSL4/LPCAT3-mediated PUFA incorporation into membranes; iron metabolism via TFR1/FPN1 regulating Fenton reaction and ROS production. **(C)** Differential ferroptosis sensitivity in bone cells: osteoblasts (OBs) and MSCs show high susceptibility with impaired differentiation; osteoclasts (OCs) exhibit bidirectional ROS response affecting NFATc1/c-Fos expression; osteocytes (OCYs) demonstrate sensitivity through Nrf2/HO-1 pathway. **(D)** Ferroptosis impact on bone homeostasis showing disrupted balance between bone formation and resorption, with potential intervention targets including SLC7A11 regulation, GPX4 stabilization, and iron chelation.

At the molecular level, ferroptosis is critically dependent on the activity of GPX4, the only known antioxidant enzyme capable of directly reducing phospholipid hydroperoxides within cellular membranes (Dos Santos and Friedmann-Angeli, 2024; Tao et al., 2025). The enzymatic function of GPX4 relies on glutathione (GSH) as an electron donor (Zhang Y. et al., 2022). When GPX4 is inactivated or when intracellular GSH is depleted, lipid peroxides accumulate within the membrane, resulting in irreversible oxidative damage (Wang et al., 2023). In parallel, free Fe^2+^ within the cytoplasm contributes to the Fenton reaction, generating hydroxyl radicals (•OH) that further propagate lipid peroxidation cascades, acting as a key driving force of ferroptosis (Liu et al., 2023; Song et al., 2024). Ferroptosis is co-regulated by multiple signaling networks, and its core regulatory mechanism can be summarized into the following functional modules (Figures 2A,B).

##### 3.1.2.1 SLC7A11-GSH-GPX4 antioxidant axis

System Xc^−^, composed of SLC7A11/SLC3A2 heterodimers, mediates the exchange of extracellular cystine for intracellular glutamate and serves as the upstream pathway for intracellular GSH synthesis (Jin et al., 2024). Downregulation of SLC7A11 expression or its functional inhibition markedly reduces intracellular GSH levels, resulting in GPX4 inactivation. This process ultimately impairs the cellular capacity to neutralize lipid peroxides, representing a critical step in the induction of ferroptosis (Yan et al., 2023).

##### 3.1.2.2 Iron metabolism and transport mechanisms

Dysregulation of iron metabolism is a crucial prerequisite for ferroptosis (Jia et al., 2024). Transferrin receptor 1 (TFR1) facilitates cellular iron uptake, ferritin heavy chain (FTH1) mediates intracellular Fe^2+^ storage, and FPN governs iron export (Bogdan et al., 2016; Deng et al., 2023). The balance between these iron regulatory proteins determines the labile iron pool within cells, which directly influences ferroptosis susceptibility.

##### 3.1.2.3 ACSL4/LPCAT3-mediated PUFA acylation and peroxidation

Long-chain acyl-CoA synthetase 4 (ACSL4) and lysophosphatidylcholine acyltransferase 3 (LPCAT3) cooperatively catalyze the acylation and incorporation of polyunsaturated fatty acids (PUFAs) into membrane phospholipids, generating molecules such as phosphatidylethanolamine-adrenic acid (PE-AdA) and phosphatidylethanolamine-arachidonic acid (PE-AA). These PUFA-containing phospholipids serve as direct substrates for iron-catalyzed lipid peroxidation (Wei et al., 2023; Dang et al., 2022). Upon GPX4 inactivation, these phospholipid PUFAs become prime targets for oxidative damage, leading to cell membrane dysfunction and the subsequent activation of ferroptotic cell death signaling.

##### 3.1.2.4 Fenton reaction-mediated oxidative toxicity

Excessive iron influx, inadequate storage capacity, or impaired export leads to accumulation of labile Fe^2+^, which enhances Fenton reaction activity, amplifies ROS production, and triggers lipid peroxidation-induced cellular damage (Chen and Chen, 2022). The Fenton reaction (Fe^2+^ + H_2_O_2_ → Fe^3+^ + •OH + OH^−^) generates highly reactive hydroxyl radicals that initiate and propagate lipid peroxidation cascades, ultimately leading to ferroptotic cell death.

### 3.2 Ferroptosis in bone homeostasis

Bone homeostasis depends on the precise coupling between osteogenesis and bone resorption (Lin et al., 2022; Huo G. et al., 2024). As a regulated way of cell death, ferroptosis shows significant heterogeneity in the response patterns of different bone-related cells (Figures 2 C,D).

#### 3.2.1 Osteoblasts

Osteoblasts dominate the synthesis and mineralization of bone matrix and are highly sensitive to iron metabolism and ROS levels (Shou et al., 2024). Studies have shown that elevated iron load or activation of ferroptosis pathway can downregulate the expression of GPX4 and SLC7A11, resulting in accumulation of lipid peroxidation and inhibition of osteoblast differentiation and mineralization (Iantomasi et al., 2023; Huo K. et al., 2024). This phenomenon suggests that the regulation of ferroptosis axis can be a new strategy for the intervention of bone formation disorders.

#### 3.2.2 Osteoclasts

Osteoclasts achieve bone renewal by absorbing bone, and are functionally opposite to osteoblasts (Liu N. et al., 2021). Different from osteoblasts, osteoclasts have a certain tolerance to ROS, and their differentiation and activity depend on the activation of ROS signaling pathway to a certain extent (Qi et al., 2024; Feng et al., 2023). It has been found that the increase of Fe^2+^ level can upregulate the expression of NFATc1 and c-Fos in osteoclast precursors and enhance their differentiation ability (Kim et al., 2019; Wang et al., 2018). However, excessive lipid peroxidation may still cause osteoclast dysfunction, suggesting that its response to ferroptosis may be bidirectional and worthy of further exploration.

#### 3.2.3 Mesenchymal stem cells (MSCs)

MSCs are precursor cells of osteoblasts, and maintaining their “stemness” is of decisive significance for bone regeneration (Zheng et al., 2022). Iron overload can induce the decrease of mitochondrial membrane potential and the increase of lipid ROS in MSCs, resulting in the decrease of pluripotency (Li M. et al., 2023; An et al., 2023). The GPX4 knockout model suggests that impaired antioxidant capacity accelerates MSCs senescence and osteogenic differentiation disorder, which is an important mechanism basis for ferroptosis-mediated bone regeneration defects (Yang et al., 2014; Bersuker et al., 2019).

#### 3.2.4 Osteocytes

As the terminal differentiation product of osteoblasts, osteocytes are the key executive units of stress perception and remodeling regulation of bone tissue (Samsa et al., 2016; Abd et al., 2018). Although the current research on ferroptosis in bone cells is limited, preliminary evidence has shown that it is highly sensitive to oxidative stress and changes in iron homeostasis, and may mediate ferroptosis response in pathological processes such as osteonecrosis and fracture repair disorders (Xu et al., 2022). The role of Nrf2/HO-1 axis in this process remains to be systematically studied and has important research potential.

In summary, ferroptosis, as a new type of programmed cell death mode, is characterized by lipid peroxidation accumulation and iron-dependent ROS burst caused by GPX4 inactivation. This process is synergistically driven by SLC7A11-GSH-GPX4 antioxidant axis, PUFA lipid acylation pathway and iron metabolism disorder. There are significant differences in the sensitivity of different types of bone-associated cells to ferroptosis: osteoblasts and MSCs are highly susceptible to ferroptosis, and their impaired function directly inhibits bone formation. Osteoclasts have a positive response to early oxidative stress signals, and may also lose bone resorption capacity when oxidative damage is excessive; as the center of bone homeostasis regulation, the role of osteocytes in ferroptosis needs to be systematically elucidated. These heterogeneous reactions not only reveal the complexity of ferroptosis in the regulation of bone homeostasis, but also provide a new perspective for understanding its bidirectional regulation in metabolic bone diseases such as osteoporosis and osteonecrosis. Future research should focus on ferroptosis threshold recognition of different osteocyte subtypes, specific molecular regulatory networks, and precise definition of intervention window period, so as to develop new anti-bone loss drugs.

## 4 Modulation of ferroptosis by Nrf2/HO-1 in bone physiology and pathophysiology

Ferroptosis is a type of programmed cell death characterized by iron-dependent lipid peroxidation accumulation. In recent years, it has been considered to play an important role in the regulation of bone homeostasis (Xiong et al., 2022; Gao et al., 2019). Nrf2 and its downstream effector HO-1 together constitute the key signal axis of cellular anti-oxidative stress and iron metabolism regulation, which not only participates in the defense of oxidative damage, but also plays a significant regulatory role in ferroptosis during the maintenance of bone metabolic balance (Montoya et al., 2021; Ma et al., 2022). Studies have shown that Nrf2/HO-1 signaling pathway is involved in the fate determination of osteoblasts and osteoclasts by regulating antioxidant defense, iron ion homeostasis and lipid metabolism, thus affecting the dynamic balance between bone formation and bone resorption (Wang N. et al., 2019; Malakoti et al., 2022).

### 4.1 Nrf2/HO-1 pathway regulates osteoblast function through ferroptosis modulation

In osteoblasts, the Nrf2/HO-1 signaling axis, as a core pathway regulating cellular antioxidant capacity and iron metabolism, is critical for inhibiting ferroptosis and protecting osteogenic function (Wu and Huang, 2024; Tonelli et al., 2018). Nrf2 transcriptionally activates a series of antioxidant enzymes, including GPX4, to effectively remove lipid peroxides in cell membrane phospholipids, thereby limiting the occurrence of ferroptosis (Fan et al., 2017; Yang et al., 2016). As the core enzyme of ferroptosis defense system, GPX4 can reduce phospholipid peroxides by GSH, which is a key factor to maintain the membrane integrity and functional stability of osteoblasts (Fang et al., 2020; Huang et al., 2024).

In addition to lipid oxidation, Nrf2 can also upregulate the expression of ferritin heavy chain 1 (FTH1), enhance the intracellular iron storage capacity, reduce the level of free Fe^2+^, and inhibit the production of hydroxyl radicals (•OH) caused by Fenton reaction, thus blocking the iron-catalyzed lipid oxidation reaction chain (Zeng et al., 2023; Liu et al., 2020). This mechanism fundamentally curbs the risk of activation of ferroptosis in osteoblasts.

**FIGURE 3:**
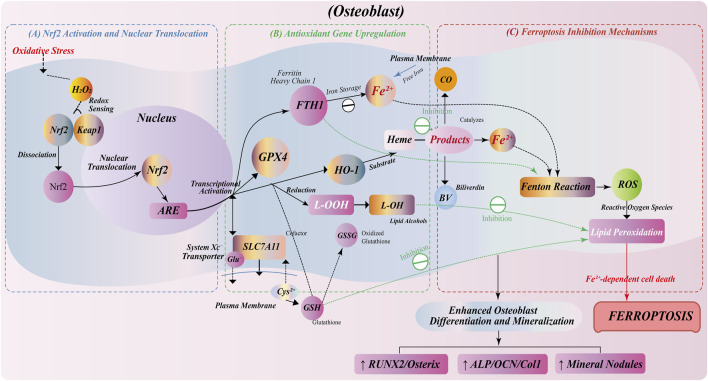
Anti-ferroptosis regulation by Nrf2/HO-1 pathway in osteoblasts. **(A)** Oxidative stress induces Nrf2 dissociation from Keap1, nuclear translocation, and ARE binding for antioxidant gene transcription. **(B)** Nrf2 activation upregulates antioxidant genes: GPX4 for lipid peroxide reduction; FTH1 for iron storage; SLC7A11 for GSH synthesis; HO-1 for heme degradation into CO, Fe^2+^, and BV. **(C)** Ferroptosis inhibition through multiple mechanisms: FTH1-mediated iron sequestration prevents Fenton reaction; GPX4/GSH system eliminates lipid peroxides; HO-1 metabolites (BV/CO) suppress ROS. This protective pathway enhances osteoblast survival, differentiation, and mineralization, with increased expression of osteogenic markers (RUNX2, Osterix, ALP, OCN, Col1) and mineralized nodule formation.

In addition, HO-1, as a classical target gene of Nrf2, catalyzes the degradation of heme to produce products-CO, BV and Fe^2+^, which can participate in anti-apoptosis and anti-inflammatory processes under certain conditions. At the same time, it cooperates with the iron homeostasis system to regulate the Fe^2+^ load level (Martínez-Casales et al., 2021; Lv et al., 2024). Although the activation of HO-1 has ferroptosis potential in some contexts, its mild induction in osteoblasts is more likely to be biased towards inhibiting lipid peroxidation and ROS accumulation, thereby exerting a protective effect (Figure 3).

In summary, the Nrf2/HO-1 pathway constructs a multi-level barrier of osteoblasts to ferroptosis stress by integrating and regulating redox homeostasis, lipid antioxidant system and iron metabolism pathway. Its activation can not only slow down cell damage and apoptosis during bone formation, but also enhance osteogenic ability and promote bone matrix deposition and mineralization, which is expected to become a potential target for maintaining bone homeostasis and preventing bone loss.

### 4.2 Bidirectional control of osteoclast activity by Nrf2/HO-1-mediated ferroptosis

The role of Nrf2/HO-1 pathway in osteoclasts shows a certain bidirectionality. Moderate Nrf2 activation has a protective effect on osteoclasts, but excessive HO-1 metabolites may promote osteoclast activity and lead to excessive bone resorption (Pan et al., 2021; Fang et al., 2024).

**FIGURE 4:**
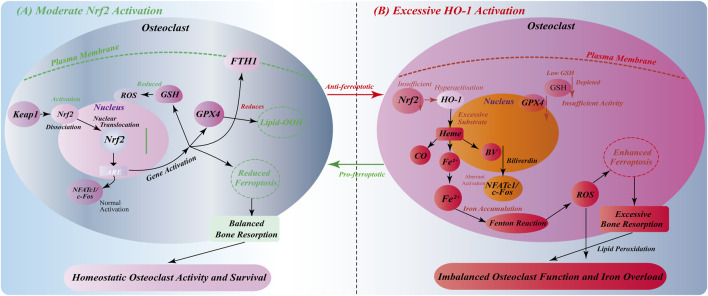
Environment-dependent bidirectional regulation of Nrf2/HO-1 signaling in osteoclasts. **(A)** Moderate Nrf2 activation: Oxidative stress induces Keap1-Nrf2 dissociation, nuclear translocation, and ARE-mediated gene expression. This upregulates GSH synthesis, GPX4 activity, and FTH1 expression, reducing ROS/lipid peroxidation and inhibiting ferroptosis. Normal NFATc1/c-Fos activation maintains balanced osteoclast function and bone resorption. **(B)** Excessive HO-1 activation: Insufficient Nrf2 function with abnormally high HO-1 expression causes excessive heme degradation and Fe^2+^ accumulation. GSH depletion and inadequate GPX4 activity, combined with Fenton reaction-generated ROS, promote ferroptosis. Iron overload triggers aberrant NFATc1/c-Fos signaling, leading to osteoclast hyperfunction and excessive bone resorption, disrupting bone homeostasis.

Moderate activation of Nrf2 can upregulate the expression of antioxidant enzymes and reduce the accumulation of ROS, thereby reducing the damage of osteoclasts caused by oxidative stress (Li et al., 2018). Oxidative stress is an important regulator of osteoclast function. Excessive ROS can cause osteoclast damage and aggravate bone resorption (Ji et al., 2023; XiaHumulus lupulus et al., 2023). Therefore, Nrf2 plays a protective role in bone metabolism by maintaining the antioxidant capacity of osteoclasts (Figure 4A).

However, excessive HO-1 metabolites, such as Fe^2+^, may have adverse effects. Ferroptosis is triggered by free radicals generated by Fenton reaction, which promote lipid peroxidation (Cai et al., 2024; Chen et al., 2023). Ferroptosis is a form of osteoclast death, and excessive Fe^2+^ can lead to excessive bone resorption (Chen Y. et al., 2024; Qu et al., 2024). Therefore, the role of Nrf2/HO-1 pathway in osteoclasts requires fine regulation. Moderate HO-1 activity has a protective effect on bone resorption, while excessive HO-1 activation may promote bone resorption and affect bone homeostasis (Figure 4B).

Taken together, this two-way regulatory mechanism reveals the importance of the precise balance of the Nrf2/HO-1 signaling axis in osteoclasts. Moderate activation has anti-ferroptosis and homeostasis maintenance effects, while excessive activation promotes ferroptosis and osteoclast dysfunction. This finding provides a new perspective for understanding the molecular mechanism of abnormal osteoclast activity in various bone metabolic diseases, and also lays a foundation for the development of precise treatment strategies for ferroptosis and bone resorption imbalance.

### 4.3 HO-1 metabolites determine osteocyte survival via ferroptosis regulation

HO-1 produces a series of metabolites by degrading heme, including CO, Fe^2+^ and BV (Tun et al., 2020). These metabolites play an important role in the regulation of bone cell fate.

**FIGURE 5:**
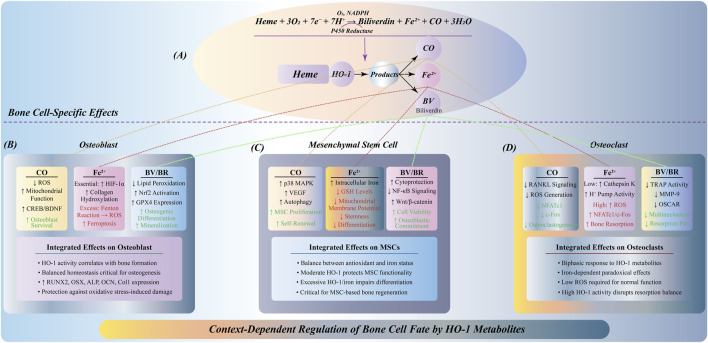
Multidimensional regulation of HO-1 metabolites on bone cell fate. **(A)** HO-1 catalyzes heme degradation to produce CO, Fe^2+^, and BV, differentially affecting three bone cell types. **(B)** In osteoblasts: CO reduces ROS and activates CREB/BDNF signaling; Fe^2+^ shows dual effects—moderate levels stabilize HIF-1α supporting bone formation, while excess induces ferroptosis; BV/BR inhibits lipid peroxidation and activates Nrf2/GPX4, promoting osteogenic differentiation. **(C)** In MSCs: CO activates p38 MAPK/VEGF promoting proliferation; Fe^2+^ accumulation depletes GSH impairing stemness; BV/BR enhances viability via NF-κB inhibition and Wnt/β-catenin activation. Dose-dependent responses are critical for MSC-based therapies. **(D)** In osteoclasts: CO inhibits RANKL signaling reducing osteoclastogenesis; Fe^2+^ effects are concentration-dependent—low levels maintain function, high levels promote excessive resorption; BV/BR suppresses TRAP/MMP-9 expression inhibiting bone resorption.

As one of the products of HO-1, CO has antioxidant properties (Zhang et al., 2020). At low concentrations, CO can protect bone cells by reducing the accumulation of ROS, improve mitochondrial function, and enhance the antioxidant capacity of cells (Wang et al., 2024). The inhibitory effect of CO on oxidative stress in osteocytes helps maintain bone homeostasis (Van Phan et al., 2013). Fe^2+^ is another important product of HO-1. Although it is essential for the physiological function of bone cells, excessive Fe^2+^ will generate free radicals through Fenton reaction, induce lipid peroxidation, and eventually lead to ferroptosis (Yang and Shang, 2022). As a form of iron-dependent cell death, ferroptosis poses a threat to the survival of bone cells. Therefore, the excessive accumulation of Fe^2+^ may have a negative impact on the health of bone cells. BV is another product produced during the degradation of heme by HO-1, which has a strong antioxidant effect (Wu and Hsieh, 2022). BV can remove excessive ROS and reduce the damage of oxidative stress to bone cells, thereby maintaining bone homeostasis (Zhou et al., 2021). Its antioxidant properties make it a key factor in protecting bone cells in HO-1 metabolites (Figure 5).

In summary, the regulation of HO-1 metabolites on the fate of osteocytes is highly environmentally dependent, and produces distinct biological effects under different cell types and different concentrations, which constitutes the molecular basis for the fine regulation of bone homeostasis. This environment-specific and cell-specific mode of action provides a new theoretical framework for understanding the pathological mechanism of bone metabolic diseases and developing targeted treatment strategies.

### 4.4 Nrf2/HO-1-ferroptosis axis in pathogenesis of bone metabolic diseases

The role of Nrf2/HO-1 pathway in bone metabolic diseases, especially in osteoporosis and osteonecrosis, has received extensive attention (Li et al., 2019; Hu et al., 2022). In these diseases, decreased expression or functional inactivation of Nrf2 is often accompanied by increased ferroptosis, excessive bone resorption, and bone loss.

**FIGURE 6:**
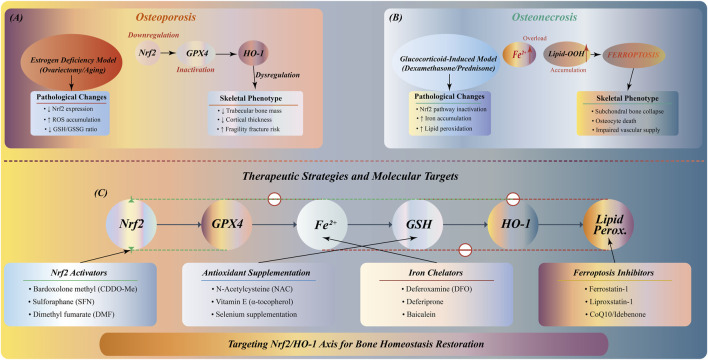
Nrf2/HO-1 pathway in bone metabolic diseases: pathology and therapeutic strategies. **(A)** Osteoporosis: Estrogen deficiency downregulates Nrf2/GPX4/HO-1, increasing ROS accumulation and decreasing GSH/GSSG ratio, resulting in trabecular bone loss, reduced cortical thickness, and fracture risk. **(B)** Osteonecrosis: Glucocorticoid treatment inactivates Nrf2 pathway, causing Fe^2+^ accumulation and lipid peroxidation, triggering ferroptosis. **(C)** Multi-target therapeutic network: Nrf2 activators (sulforaphane, melatonin), antioxidants (vitamin E, quercetin), iron chelators (DFO, deferiprone), and ferroptosis inhibitors (ferrostatin-1) targeting different nodes of the pathway.

In the animal model of estrogen deficiency-induced osteoporosis, the expression of Nrf2 is significantly decreased, which is closely related to the inhibition of osteogenesis and the decrease of bone mineralization function (Liu et al., 2024). Studies have shown that Nrf2 activation can upregulate the expression of antioxidant enzymes, reduce oxidative stress and ferroptosis, thereby inhibiting bone resorption, promoting bone formation, improving bone structure, and slowing the process of osteoporosis and osteonecrosis (Xue et al., 2019). In addition, HO-1, as a downstream effector molecule of the Nrf2 pathway, its inducer can promote bone repair by reducing ROS accumulation and inhibiting bone resorption in the early stage (Han et al., 2022; Liu et al., 2025). Therefore, the regulation of Nrf2/HO-1 pathway has potential application prospects in the treatment of bone metabolic diseases such as osteoporosis and osteonecrosis (Figure 6).

Therefore, Nrf2 dysfunction caused by estrogen deficiency or glucocorticoid treatment leads to increased oxidative stress, iron metabolism disorders and lipid peroxidation, and ultimately damages bone structure. Based on these findings, multi-target treatment strategies from Nrf2 activators to ferroptosis inhibitors have shown clinical application prospects. However, there are still many challenges in this field. The regulatory mechanism of Nrf2/HO-1 pathway in different bone cells has not been fully elucidated. The targeted delivery and long-term safety of therapeutic drugs need to be verified, and the interaction with other bone metabolic networks needs further study.

## 5 Therapeutic targeting of the Nrf2/HO-1-ferroptosis axis in bone diseases

The Nrf2/HO-1 pathway plays a pivotal role in regulating bone homeostasis through modulation of ferroptosis, providing novel therapeutic strategies for various bone diseases (Zhang et al., 2023). This section discusses targeted interventions based on specific bone pathologies, highlighting how manipulation of the Nrf2/HO-1-ferroptosis axis can effectively improve disease outcomes (Table 1).

**TABLE 1:** Therapeutic interventions targeting the Nrf2/HO-1-ferroptosis axis in bone diseases: Mechanisms and clinical applications.

Disease category		Compound	Main mechanism	Clinical application	References
Osteoporosis	Postmenopausal	Sulforaphane	Alkylation of Keap1 Cys151→Nrf2/ARE activation↑→↑HO-1, NQO1 expression	OVX rats: ↑BMD and improved trabecular architecture	Thaler et al. (2016)
	Diabetic	Melatonin	MT2→ERK/AKT→↑Nrf2/HO-1→enhanced ROS clearance	Improved bone mineral density	Hu et al. (2022)
	General	α-Tocopherol (Vitamin E)	Lipid-chain termination→radical clearance→reinforced membrane antioxidant barrier	Prevention: improved implant biocompatibility	Lovati et al. (2018)
	General	Quercetin	PI3K/AKT→↑Nrf2+↓IKK/NF-κB→dual antioxidant and anti-inflammatory effects	Enhanced osteoblast mineralization	Mao et al. (2024)
Osteonecrosis	Glucocorticoid-induced	Deferoxamine	Fe^2+^ chelation→Fenton reaction blockade→↑HIF-1α stabilization→↑VEGF release	Bone defect scaffolds: ↑vascularization and osteogenesis	Shen et al. (2024)
	Bone defects	Curcumin	p62-mediated Keap1 degradation→↑Nrf2↓JAK/STAT→anti-inflammatory	Nanoparticle delivery: enhanced bone defect repair	Astaneh et al. (2024)
	General	Ferrostatin-1	Inhibition of ACSL4-mediated PUFA peroxidation→mitochondrial membrane protection	*In vitro* MSCs: ↑viability	Valanezhad et al. (2021)
Inflammatory Bone Diseases	Osteolytic conditions	Dimethyl fumarate	Keap1 alkylation→ARE activation↑→↑GSH synthesis→ferroptosis inhibition	Osteolytic models: ↓osteoclast activity, ↑Runx2	Yamaguchi et al. (2018)
	Rheumatoid arthritis	Tin protoporphyrin IX	Competitive HO-1 inhibition→↓CO production→suppress excess HO-1-mediated bone resorption	Inhibited bone loss	Ibáñez et al. (2011)
	Osteoarthritis	Quercetin	PI3K/AKT→↑Nrf2+↓IKK/NF-κB→dual antioxidant and anti-inflammatory effects	Cartilage protection	Mao et al. (2024)
	Radiation-induced injury	Tanshinone IIA	SIRT1-mediated Nrf2 deacetylation→↑ARE-driven antioxidants↓p38/MAPK→inflammation inhibition	Restoration of marrow microenvironment	Cheng et al. (2024)
Iron Overload Disorders	Transfusion-induced	Deferiprone	Small-molecule chelation of labile iron→↓ROS	Bone density preservation	Shen et al. (2024)
	Chronic overload	Deferasirox	Bidentate Fe^3+^ coordination→↓bone-matrix iron accumulation	Reduced fracture risk	Casale et al. (2014)
Emerging Therapies	Bardoxolone methyl (CDDO-Me)		Keap1 Cys151 binding→ultra-potent Nrf2 activation↓NF-κB→potent antioxidant/anti-inflammatory	Phase I trial: potential trabecular bone protection	Yang et al. (2023)

### 5.1 Therapeutic strategies for osteoporosis

Osteoporosis, characterized by decreased bone density and increased fracture risk, has emerged as a primary target for Nrf2/HO-1 pathway modulation. Nrf2 agonists have shown remarkable efficacy in preclinical osteoporosis models. Sulforaphane, through alkylation of Keap1 Cys151 residues, promotes Nrf2 nuclear translocation and ARE activation, leading to increased HO-1 and NQO1 expression (Lin et al., 2014). In ovariectomized rat models, sulforaphane treatment significantly improved BMD and trabecular architecture (Thaler et al., 2016).

Melatonin represents another promising therapeutic agent for diabetic osteoporosis. By activating the MT2-ERK/AKT-Nrf2/HO-1 cascade, melatonin reduces ROS levels, upregulates SLC7A11 expression, and increases GPX4 activity, thereby protecting osteoblasts from ferroptosis and improving bone mineral density (Hu et al., 2022) (Yan et al., 2022). Similarly, traditional Chinese medicine components have demonstrated efficacy: quercetin enhances osteoblast mineralization through PI3K/AKT-mediated Nrf2 activation, while simultaneously suppressing NF-κB inflammatory signaling (Xiao et al., 2023; Mao et al., 2024).

For postmenopausal osteoporosis, α-tocopherol (Vitamin E) functions as a lipid-chain termination agent, reinforcing membrane antioxidant barriers and improving implant biocompatibility (Lovati et al., 2018). These diverse approaches underscore the multifaceted therapeutic potential of targeting the Nrf2/HO-1 pathway in osteoporosis management.

### 5.2 Interventions for osteonecrosis

Osteonecrosis, particularly glucocorticoid-induced osteonecrosis, involves excessive ferroptosis and compromised bone vascularization. Iron chelators have emerged as crucial therapeutic agents in this context. Deferoxamine (DFO) blocks the Fenton reaction by chelating Fe^2+^, leading to HIF-1α stabilization and enhanced VEGF release, thereby promoting vascularization and osteogenesis in bone defect models (Guo et al., 2023; Shen et al., 2024).

The nano-delivery system has shown particular promise for osteonecrosis treatment. Curcumin-loaded nanoparticles enhance bone defect repair through p62-mediated Keap1 degradation, resulting in sustained Nrf2 activation and dual antioxidant/anti-inflammatory effects (Wang, 2024; Astaneh et al., 2024). This targeted delivery approach improves bioavailability while minimizing systemic side effects (Cheng et al., 2017; Chen Y. J. et al., 2024).

Combined therapeutic strategies have proven especially effective. The synergistic use of DFO with lipid antioxidants like Ferrostatin-1 addresses both iron overload and lipid peroxidation, providing comprehensive protection against ferroptotic cell death in osteonecrosis (Guo et al., 2023; Valanezhad et al., 2021).

### 5.3 Applications in inflammatory bone diseases

Inflammatory bone diseases, including rheumatoid arthritis and osteoarthritis, present unique therapeutic challenges due to the interplay between inflammation and oxidative stress. Dimethyl fumarate targets this dual pathology through Keap1 alkylation, promoting GSH synthesis while inhibiting ferroptosis. In osteolytic models, this approach reduces osteoclast activity and upregulates Runx2 expression (Sánchez-de-Diego et al., 2021; Yamaguchi et al., 2018).

For rheumatoid arthritis, HO-1 modulation requires careful balance. Tin protoporphyrin IX, a competitive HO-1 inhibitor, reduces CO production and suppresses excess HO-1-mediated bone resorption, effectively inhibiting bone loss in arthritis models (Ibáñez et al., 2011). This highlights the importance of context-dependent HO-1 regulation (Kajarabille and Latunde-Dada, 2019; Li et al., 2020; Yang et al., 2022; Castany et al., 2016).

Tanshinone IIA offers a multifaceted approach for radiation-induced bone injury through SIRT1-mediated Nrf2 deacetylation, driving ARE-dependent antioxidant expression while suppressing p38/MAPK inflammatory signaling. This dual action helps restore bone marrow microenvironment (Cao et al., 2024; Cheng et al., 2024).

### 5.4 Treatment of iron overload-related bone disorders

Chronic iron overload conditions, such as those seen in transfusion-dependent thalassemia, require specialized therapeutic approaches. Deferiprone provides small-molecule chelation of labile iron, effectively preserving bone density in transfusion-induced iron overload (Shen et al., 2024). Similarly, deferasirox coordinates bidentate Fe^3+^ binding to reduce bone-matrix iron accumulation, significantly decreasing fracture risk in chronic iron overload patients (Casale et al., 2014).

These iron-specific interventions demonstrate the critical importance of maintaining iron homeostasis in bone health, particularly in patients with systemic iron metabolism disorders.

### 5.5 Emerging therapeutic modalities and future directions

Novel therapeutic agents continue to emerge. Bardoxolone methyl (CDDO-Me), currently in Phase I trials, represents an ultra-potent Nrf2 activator through Keap1 Cys151 binding, offering potential trabecular bone protection with combined antioxidant and anti-inflammatory properties (Yang et al., 2023).

Advanced delivery systems are revolutionizing treatment approaches. Bone-targeted nanoparticle formulations enable precise delivery of Nrf2 agonists or traditional medicine components directly to affected bone tissue, maximizing therapeutic efficacy while minimizing off-target effects (Astaneh et al., 2024; Cheng et al., 2017; Chen Y. J. et al., 2024).

The integration of combination therapies—utilizing Nrf2 activators (Xiao et al., 2023; Cao et al., 2024), iron chelators (Guo et al., 2023), and ferroptosis inhibitors (Valanezhad et al., 2021)—represents the future of personalized bone disease treatment, allowing clinicians to address the specific pathological mechanisms underlying each patient’s condition.

## 6 Conclusion and prospects

Whether ferroptosis constitutes an indispensable driver of osteonecrosis or osteoporosis remains an open question. Although Fe^2+^ accumulation, lipid peroxidation, and the ensuing oxidative stress have been consistently observed in diverse bone‐pathology models—most notably in glucocorticoid‐induced osteoporosis and osteonecrosis—definitive proof that ferroptosis is a “necessary condition” for disease initiation is still lacking. It is increasingly apparent that ferroptosis may represent a critical facet of disease progression rather than the singular pathogenic mechanism. Adding further complexity, the Nrf2 signaling axis exerts dichotomous effects: on one hand, activation of Nrf2 promotes the transcription of antioxidant enzymes, mitigates oxidative damage, and suppresses ferroptosis, thereby safeguarding osteocytic integrity; on the other hand, chronic or excessive Nrf2 activation can drive overproduction of HO-1 metabolites (including labile Fe^2+^), thereby potentiating ferroptotic cell death and skeletal deterioration. Dissecting these “protective” versus “pathogenic” roles of Nrf2—particularly in a cell‐type–and time‐dependent manner (e.g., osteoblasts versus osteoclasts, early versus late disease stages)—remains a formidable challenge. Moreover, the translational gap between animal models and human clinical specimens is still wide: while preclinical data are abundant, robust validation in patient cohorts is insufficient. Future research must therefore integrate sophisticated *in vivo* and *ex vivo* platforms, and prioritize the development of bone‐targeted, tissue‐specific Nrf2 modulators capable of finely tuning redox homeostasis without eliciting systemic iron overload.

Overall, the Nrf2/HO-1 signaling cascade emerges as a pivotal regulator of bone homeostasis, intricately linking oxidative stress, iron metabolism, and regulated cell death pathways. Investigations into ferroptosis have unveiled novel insights into the mechanistic interplay between iron dysregulation and osteocellular injury, offering a fresh conceptual framework for osteoporosis and osteonecrosis pathogenesis. Looking ahead, the precise manipulation of the Nrf2/HO-1 axis—particularly through bone‐targeted, cell‐contextual therapeutics—holds promise for transformative interventions in skeletal disease. Realizing this potential will require surmounting key obstacles, including delineation of cell‐type specificity, temporal window optimization, and the refinement of delivery systems to ensure localized efficacy while minimizing off‐target effects. With continued multidisciplinary advances, targeted modulation of the Nrf2/HO-1 pathway is poised to inaugurate a new era of precision medicine in bone pathology.
